# Pancreas-visceral fat CT value ratio and serrated pancreatic contour are strong predictors of postoperative pancreatic fistula after pancreaticojejunostomy

**DOI:** 10.1186/s12893-020-00785-w

**Published:** 2020-06-11

**Authors:** Tomoki Kusafuka, Hiroyuki Kato, Yusuke Iizawa, Daisuke Noguchi, Kazuyuki Gyoten, Aoi Hayasaki, Takehiro Fujii, Yasuhiro Murata, Akihiro Tanemura, Naohisa Kuriyama, Yoshinori Azumi, Masashi Kishiwada, Shugo Mizuno, Masanobu Usui, Hiroyuki Sakurai, Shuji Isaji

**Affiliations:** Department of Hepatobiliary Pancreatic and Transplant Surgery, Mie University Graduate School of Medicine, 2-174 Edobashi, Tsu, Mie 514-8507 Japan

**Keywords:** BMI, Albumin, Pancreatic parenchymal CT value, Visceral fat CT value

## Abstract

**Background:**

Our aim is to elucidate the true preoperative risk factors for postoperative pancreatic fistula (POPF) after pancreaticoduodenectomy (PD), making it possible to select POPF high-risk patients preoperatively regardless of intraoperative pancreatic consistency judged by the surgeon’s hand.

**Methods:**

Among the 298 patients who underwent PD with pancreaticojejunostomy from 2007 to 2016, 262 patients had preoperative CT configurations that could be precisely evaluated. Risk factor analyses were conducted using various perioperative factors, including preoperative CT findings, such as CT values of the pancreas, pancreas-visceral fat CT value ratio and pancreatic outer contour. Pancreatic outer contour was further divided into smooth- (smooth interlobular) and serrated-type contours (feathery, irregular interlobular) by preoperative CT.

**Results:**

In terms of the incidence of POPF, among the 262 patients, POPF grade B/C was found in 27 (10.3%): grade B in 23 (8.8%) and grade C in 4 (1.5%). According to multivariate analysis, a high pancreas-visceral fat CT value ratio (*p* = 0.002), serrated-type contour (*p* = 0.02) and no history of chemoradiotherapy (*p* = 0.019) were identified as independent risk factors for POPF grade B/C. Even in patients with soft pancreas, the incidence of POPF grade B/C was 0% (0/57) in patients with a pancreas-visceral fat CT value ratio of less than − 0.4 and smooth-type contour, whereas the incidence was markedly high (45.0%, 9/20) in patients with a pancreas-visceral fat CT value ratio of − 0.4 or greater and serrated-type contour, indicating that patients with soft pancreas should be categorized into POPF high-risk and low-risk groups according to preoperative CT scan results.

**Conclusions:**

The pancreas-visceral fat CT value ratio and serrated-type pancreas are useful markers to preoperatively identify true POPF high-risk groups in patients undergoing PD, regardless of the pancreatic texture judged intraoperatively.

## Background

The probability of postoperative in-hospital mortality after pancreatoduodenectomy (PD) has decreased, especially in high-volume centres, with a mortality rate of less than 4% over recent decades [1, 2]. A recent study using a national clinical database from Japan revealed that the 30-day and in-hospital mortality rates were 1.2 and 2.8%, respectively [3]. Despite the fact that a low mortality rate has been observed, the incidence of clinically relevant postoperative pancreatic fistula (POPF: grade B/C), which most negatively affects patient outcome, has been recently reported to be 11–37% in patients with soft pancreas and 1–6% in patients with hard pancreas [4–9]. Regarding the risk factors for POPF, previous studies have reported various risk factors, such as age, sex, preoperative jaundice, operative time, intraoperative blood loss, type of pancreatic reconstruction, anastomotic technique, consistency of the pancreatic stump and pancreatic duct diameter [10–14], but there have been no reports focusing on preoperative computed tomography (CT) configurations, especially the contour of the pancreas, for predicting POPF preoperatively.

The procedures of pancreatoenteral anastomosis have not been standardized, and each institution performs their own preferred procedure, such as pancreaticogastrostomy, pancreaticojejunostomy, external tube drainage, the lost stent method and invagination; this diversity of procedures makes it difficult to evaluate the frequency of POPF [15–17]. Our institution reported the method of 12 interrupted-stitched duct-to-mucosa pancreaticojejunostomy, named the “pair-watch suturing technique (PWST)”, which allowed us to standardize the method of pancreaticojejunostomy [18–20]. However, even though the anastomotic technique has progressed, POPF still has yet to be thoroughly prevented after PD, and the incidence of POPF in patients with soft pancreas has been reported to be particularly high; thus, the prevention of POPF in patients with soft pancreas is still under discussion [21–23].

Recently, Sugimoto M et al. [21] reported that a thick parenchyma, a small main pancreatic duct (MPD), and fatty infiltration determined by postoperative histology were strongly associated with clinically relevant POPF after PD, especially in patients with soft pancreas, which was judged by intraoperative findings, and the study showed the negative impact of fat infiltration into the pancreatic parenchyma. Because pancreatic texture and consistency can be determined only by intraoperative findings or postoperative histological examinations, a high-risk group of POPF patients, especially those with soft pancreas, cannot be identified preoperatively. To solve this problem, Kuwahara T et al. [24] showed the usefulness of preoperative endoscopic ultrasonography-elastography (EUS-EG), which made it possible to objectively assess tissue elasticity preoperatively and predict the development of POPF after PD. However, EUS-EG is still an uncommon procedure for the preoperative assessment of pancreatic consistency, and we consider it indispensable to select true soft pancreas and POPF high-risk patient groups preoperatively based on pancreatic configurations, such as the MPD diameter and parenchymal thickness, and on CT attenuation values of the pancreas, such as visceral fat and other ratios. All these parameters are easily measurable by plain CT images.

In terms of the contour of the pancreas in preoperative CT, the precise cause of significant changes in the irregularity of the borders of the pancreas is unknown. When we analysed CT images to evaluate the precise morphology of the pancreas in order to investigate the type of pancreas that is likely to develop POPF, we noticed that 20 to 30% of patients with a mostly normal pancreas had irregular pancreatic borders, so we called this type of pancreas a serrated pancreas. According to previous research, a serrated pancreatic border is reported to be the result of ageing or acute weight loss after reversal of type 2 diabetes mellitus (DM) treated by a low-calorie diet [25]. This report also showed that the pancreas of patients with type 2 DM obviously had more pancreatic marginal irregularities compared with the pancreas of healthy patients. In the field of diabetic internal medicine, pancreatic contour has been sometimes discussed, but there have been no studies evaluating the relationship between pancreatic outer contour and POPF after PD. Moreover, we hypothesized that a serrated pancreatic contour was associated with intralobular frailty, which results in parenchymal vulnerability during pancreaticojejunostomy. This vulnerability might result in difficulties associated with the anastomosis, with determining the risk of POPF regardless of the MPD size and pancreatic thickness, and with surgeons anastomosis skills.

In the present study, we evaluated 262 patients who underwent PD with PWST and analysed the pre- and intraoperative risk factors for POPF in these patients, focusing on the association between the incidence of POPF and the preoperative CT configuration of the pancreas as well as the CT attenuation values of the pancreatic parenchyma, visceral fat and these ratios. Our aim was to elucidate the true preoperative risk factors for POPF even in patients with soft pancreas, making it possible to select POPF high-risk patients preoperatively regardless of intraoperative pancreatic consistency judged by the surgeon’s hand.

## Methods

### Patients

**Fig. 1:**
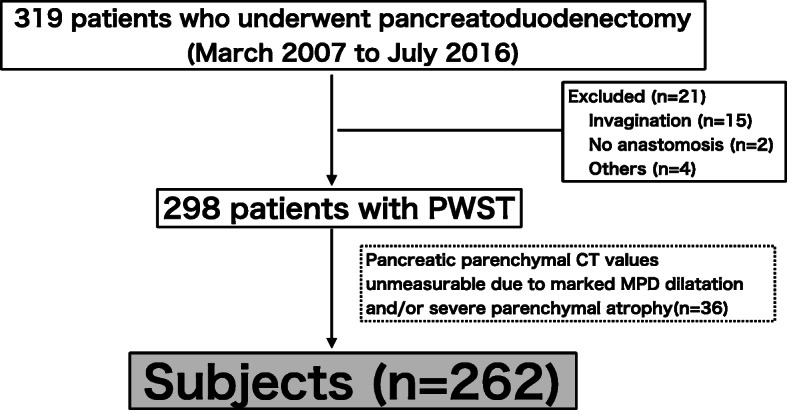
Flow diagram of the subjects for the study. POPF: postoperative pancreatic fistula, PWST: pair-watch suturing technique, PDAC: pancreatic ductal adenocarcinoma, MPD: main pancreatic duct

Among 319 patients who underwent PD from April 2007 to December 2016, PWST was performed in 298; there were 262 patients in the present study in whom the preoperative CT configuration could be precisely evaluated (Fig. 1). We retrospectively analysed the perioperative factors of POPF grade B/C, including various preoperative CT configurations. The study protocol was approved by the medical ethics committee of Mie University Hospital (No. 2857), and the study was performed in accordance with the ethical standards established in the 1964 Declaration of Helsinki.

### Surgical procedure

For pancreaticojejunostomy, the first-layer anastomosis, which was a duct-to-mucosa anastomosis, was performed using PWST with 6–0 PDS II (Ethicon, Inc. Somerville, NJ, USA). This technique was conducted using 12 interrupted sutures oriented in a clock formation regardless of the MPD diameter [18–20]. This can be imagined as the faces of a pair of wristwatches, with the jejunal hole corresponding to the left-hand watch and the pancreatic duct hole to the right-hand one. The posterior wall of the pancreatic duct consists of the latter half of the clock cycle, from 6 to 12 o’clock, and the posterior wall of the jejunal hole consists of the first half of the clock cycle, from 12 to 6 o’clock. The second-layer anastomosis was a pancreatic parenchymal-jejunal seromuscular anastomosis, which was performed via interrupted sutures with 4–0 Vicryl. In this study, PWST was carried out in all 262 patients. The surgical procedures included conventional PD in 28 patients, pylorus-preserving PD (PPPD) in 6 patients and subtotal stomach-preserving PD (SSPPD) in 228 patients. Laparoscopic SSPPD was performed in 12 patients, all of whom underwent the reconstruction procedures of pancreaticojejunostomy and hepaticojejunostomy under mini-laparotomy. Reconstruction was carried out via a modified Child’s method. A 5 Fr pancreatic stent tube was placed in patients with soft pancreas and/or a narrow MPD according to the surgeon’s decision. A feeding jejunostomy tube was placed intraoperatively for early postoperative enteral nutrition. A single abdominal drain was inserted through the foramen of Winslow near the site of pancreaticojejunostomy. A drain was removed until postoperative day (POD) 5, as long as the drain discharge was clear and the drain amylase level was not three times higher than the upper limit of the serum amylase level (132 U/ml). A somatostatin analogue was not prophylactically used for preventing POPF.

### Assessment of POPF

POPF was defined and graded according to the International Study Group on Pancreatic Fistula classification [26]. In all patients, the amylase activities of the abdominal drainage fluid were measured on postoperative day (POD) 3 to 7. For the diagnosis of POPF, any measurable volume of drainage fluid with an amylase level > 3 times the upper limit of normal amylase (132 U/l) was considered the necessary threshold. POPF without any specific treatment despite the high drainage amylase level was categorized as biochemical leakage (BL). POPF was categorized as grade B when patients needed the following treatments: persistent drainage for more than 3 weeks, clinically relevant drain exchange, percutaneous or endoscopic drainage, and angiographic procedures. POPF was defined as grade C when reoperation was performed or when organ failure developed due to POPF.

In this study, we focused on clinically relevant grade B/C POPF. To identify the pre- and intraoperative risk factors for POPF, we compared various factors between patients with non-POPF or BL and those with POPF grade B/C.

### Measurements of CT attenuation values of the pancreatic parenchyma and visceral fat

**Fig. 2:**
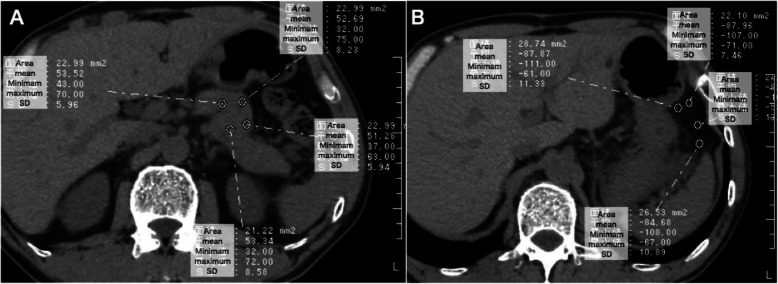
Representative images for the measurements of CT values of the pancreatic parenchyma (**a**) and visceral fat (**b**). **a** Pancreatic CT values in the future remnant pancreatic body-tail are measured in the four different ROIs area of 15 to 30 mm2 on a magnified CT image. **b** Visceral fat CT values at lateral to the stomach are measured in the four different ROIs area of 15 to 30 mm2. ROI: region of interest, SD: standard deviation

First, we conjecture that a lower CT attenuation value of the pancreatic parenchyma reflects fat deposition into the pancreas, a lower CT attenuation value of visceral fat reflects adipose tissue hypertrophy, and these ratios might represent parenchymal quality. Thus, we measured these values and evaluated the association between these values and POPF grade B/C. Pancreatic parenchymal CT attenuation values in the future remnant pancreatic body/tail were measured at four different areas whose regions of interest (ROIs) were set by dragging the desired round area of 15 to 30 mm^2^ on a magnified CT image (Fig. 2a). To obtain accurate reproducibility, we concurrently used dynamic CT scans, including the arterial, portal and equilibrium phases, to exclude the areas of the pancreatic duct, splenic artery, splenic vein, portal vein and superior mesenteric artery. Visceral fat CT attenuation values were measured lateral to the stomach in four different areas whose ROIs were 15 to 30 mm^2^ (Fig. 2b). The mean CT value of the 4 different points of ROIs was employed for each CT scan. The pancreas-visceral fat CT value ratio was calculated as the mean pancreatic parenchymal CT value/mean visceral fat CT value. The MPD diameter was measured on CT at the planned resection level, and the pancreatic parenchymal thickness at the planned resection level was calculated by the following formula: the thickness of the pancreas (mm) - MPD diameter (mm).

### Configuration of the pancreatic outer contour determined by preoperative plain CT scan

**Fig. 3:**
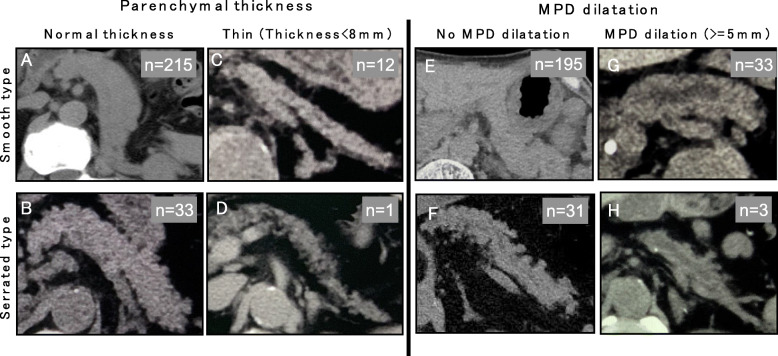
Morphology and contour of pancreas preoperative plain CT. We divided the pancreatic CT configuration into a smooth type (Upper lane) and serrated type (lower lane). **a.** Smooth type in the pancreas with normal thickness. **b.** Serrated type in the pancreas with normal thickness. **c.** Smooth type in the thin pancreas. **d.** Serrated type in the thin pancreas. **e.** Smooth type without dilatation of MPD. **f.** Serrated type without dilation of MPD. **g.** Smooth type with dilation of MPD H. Serrated type with dilation of MPD MPD: main pancreatic duct

To determine the significant morphology of the pancreas influencing the development of POPF, all 262 pancreatic margins were traced, and we categorized the pancreatic contour into smooth- and serrated-type contours. According to the plain CT scans, the smooth type was defined as a pancreas with a smooth interlobular border, and the serrated type was defined as a pancreas with a feathery, irregular interlobular border and with a protrusion shape of more than 3 mm, as shown in Fig. 3. Regardless of the pancreatic configuration, such as the thickness and presence or absence of MPD dilatation, a serrated-type pancreas was found in all categories of pancreatic configurations, but it was more frequently seen in the normal pancreas than in the atrophic pancreas and/or the pancreas with MPD dilatation.

### Risk factor analysis for POPF

Uni- and multivariate analyses were conducted to evaluate the risk factors for POPF grade B/C using pre- and intraoperative factors individually. The preoperative factors included age, sex, PDAC status, diabetes mellitus, Body mass index (BMI), history of chemoradiotherapy, MPD diameter on CT (mm), parenchymal thickness on CT (mm), pancreatic parenchymal CT value (Hounsfield units; HU), visceral fat CT value (HU), pancreas-visceral fat CT value ratio and type of parenchymal contour. The intraoperative factors included the type of procedure, the use of laparoscopic surgery, combined portal vain (PV) resection, combined artery resection, combined distal pancreatectomy, pancreatic texture, MPD diameter judged intraoperatively (mm), presence or absence of pancreatic stent tube, operation time, and intraoperative blood loss (ml).

### Statistical analysis

All statistical analyses were performed using the statistical software package SPSS for Macintosh (version 24.0, IBM, Armonk, NY, USA). The results of the continuous variables were expressed as the median and range, and statistical significance was determined by the Mann-Whitney U test. Discrete variables were evaluated by χ^2^ analysis or Fisher’s exact test, as appropriate. The pre- and intraoperative risk factors associated with POPF were analysed by uni- and multivariate analyses (multi-regression analysis). Only variables whose *p*-values were less than 0.05 according to univariate analysis were included in the multivariate analysis. The results were considered significant when the p-values were less than 0.05. The optimal cut-off value of the pancreas-visceral fat CT value ratio was determined using the diagnostic accuracy measurements and the receiver-operating characteristic (ROC) curves and was calculated on the basis of the maximum values of the Youden index, calculated by [sensitivity + specificity – 1].

## Results

The characteristics of the subjects are shown in Table 1. In these 262 patients, the median age (range) was 67.6 years old, and the male/female ratio was 158/104. The primary diseases were pancreatic adenocarcinoma (PDAC) in 118 patients, intraductal papillary mucinous neoplasm (IPMN) in 52 patients, bile duct carcinoma in 53 patients and others in 39 patients. The results of the CT analysis were as follows: MPD diameter (mm) = 3.0, pancreatic parenchymal thickness (mm) = 13.5, pancreatic parenchymal CT value (Hounsfield Unit: HU) = 38.2, visceral fat CT value (HU) = − 98.2, and pancreas-visceral fat CT value ratio = − 0.39, and the pancreatic texture judged intraoperatively was soft in 123 (46.9%) and hard in 139 patients (53.1%).

**Table 1:** Characteristics of the patients undergoing PD with PWST

Patient’s background	*n* = 262
Age	67.6 (25–91)
Gender: M / F	158/104
Diagnosis: PDAC/IPMN/bile duct cancer/others	118/52/53/39
Preoperative diabetes mellitus (yes/no)	87/175
BMI (kg/m2)	22.2 (14.1–40.0)
Chemoradiotherapy (yes/no)	98/164
MPD diameter on CT (mm)	3.0 (1.0–12.6)
Pancreatic parenchymal thickness on CT *(mm)	13.5 (4.5–27.0)
Pancreatic parenchymal CT value	38.2 (9.7–56.5)
Visceral fat CT value	−98.2 (− 123.1 ~ −23.0)
Pancreas-visceral fat CT value ratio	− 0.39 (− 1.21 ~ − 0.06)
Procedure: PPPD/SSPPD/PD	6/228/28
Surgeon’s experience: < 20 cases / 20 = < cases	180/82
Laparoscopic surgery (yes/no)	12/250
Combined PV resection (yes/no)	111/151
Pancreatic texture judged intraoperatively (soft/hard)	123/139
Pancreatic stent (yes/no)	140/122
Operation time (min)	526 (286–1373^a^)
Intraoperative blood loss (ml)	863 (20–20,983^b^)
Type of pancreatic contour (smooth / serrated)	228/34
POPF: non/BL/B/C	215/20/23/4

Parenchymal thickness = the thickness of the pancreas (mm) - MPD diameter (mm), at the planned cut line

^a^This case underwent SSPPD, transverse colectomy and low anterior resection for triple cancer (duodenal papilla, transverse colon and rectum)

^b^This case developed intraoperative massive bleeding due to the presence of intraabdominal abscess and severe adhesion to adjacent organs and vessels, but finally recovered

*PDAC* pancreatic ductal adenocarcinoma, *IPMN* intraductal papillary mucinous neoplasm, *BMI* body mass index, *MPD* main pancreatic duct, *PPPD* pylorus-preserving pancreaticoduodenectomy, *SSPPD* subtotal stomach-preserving pancreaticoduodenectomy, *PD* pancreaticoduodenectomy, *PV* portal vein, *POPF* postoperative pancreatic fistula, *BL* biochemical leak

In terms of the type of pancreatic outer contour, the smooth type was observed in 228 (87%) and the serrated type in 34 patients (13%).

A pancreatic stent was placed in 140 (53.4%) patients. The operation time (min) and intraoperative blood loss (ml) were 526 and 863, respectively. In terms of the incidence of POPF, among the 262 patients, clinically relevant POPF, that is, POPF grade B/C, was found in 27 patients (10.3%): grade B in 23 (8.8%) and grade C in 4 (1.5%). For reference, BL was found in 20 patients (7.6%). In terms of the treatment of POPF grade B, CT-guided drainage was performed in 10 patients, re-initiation of antibiotics in 4, wound drainage in 3, drain exchange in 3, angiography for bleeding in 2 and persistent drainage for more than 3 weeks in one. In the 4 patients with POPF grade C, open laparotomy was performed in 3 patients, and mechanical ventilation for the treatment of acute lung injury was performed in one patient.

### Pre- and intraoperative risk factors for POPF

As shown in Table 2, univariate analysis performed by comparing the preoperative risk factors between the POPF grade B/C group and the non-POPF, BL group identified the following significant factors: male sex (*p* = 0.050), non-PDAC (*p* = 0.029), higher BMI (*p* = 0.002), absence of a history of chemoradiotherapy (*p* = 0.010), higher pancreatic parenchymal CT value (*p* = 0.028), lower visceral fat CT value (HU), higher pancreas-visceral fat CT value ratio (*p* = 0.00025), and serrated-type contour (*p* < 0.001). According to multivariate analysis, a higher pancreas-visceral fat CT value ratio (*p* = 0.002) and frequency of serrated-type contour (*p* = 0.020) and the lack of a chemoradiotherapy history (*p* = 0.019) were calculated as independent risk factors for POPF.

**Table 2:** Univariate and multivariate analysis for evaluating preoperative risk factors associated with POPF

Variables	non-POPF, BL (***n*** = 235)	POPF Grade BC (***n*** = 27)	***P***-value (Univariate)	Odd’s ratio (95% CI)	***P***-value (Multivariate)
**Age**	**67.0 (25–89)**	**67.0 (25–89)**	**0.094**		
**Gender: M / F**	**137/98**	**21/6**	**0.050**	**–**	**–**
**Diagnosis: (PDAC/non-PDAC)**	**113/122**	**7/20**	**0.029**	**–**	**–**
**Preoperative diabetes mellitus (yes/no)**	**81/154**	**6/21**	**0.201**		
**BMI (kg/m2)**	**21.1 (14.9–40.0)**	**23.7 (14.1–31.0)**	**0.002**	**–**	**–**
**Chemoradiotherapy (yes/no)**	**94/141**	**4/23**	**0.010**	**0.25 (0.08–0.80)**	**0.019**
**MPD diameter on CT (mm)**	**3.0 (1.0–13.6)**	**2.5 (1.0–6.0)**	**0.160**		
Pancreatic parenchymal thickness on CT *(mm)	**13.5 (4.5–27.0)**	**14.0 (25.0–25.0)**	**0.348**		
**Pancreatic parenchymal CT value (HU)**	**38.5 (10.2–56.5)**	**35.8 (9.7–54.8)**	**0.028**	**–**	**–**
**Visceral fat CT value (HU)**	**−97.5 (−123.1- − 100.8)**	**-100.8 (− 120.7 - -23.0)**	**0.025**	**–**	**–**
**pancreas-visceral fat CT value ratio**	**−0.40 (−1.21- -0.117)**	**−0.35 (−0.42–0.09)**	**0.00025**	**2891.5 (17.6–473,225.1)**	**0.002**
**Type of parenchymal contour (Serrated/smooth)**	**24/211**	**10/17**	**< 0.001**	**3.11 (1.20–8.06)**	**0.020**

Parenchymal thickness = the thickness of the pancreas (mm) - MPD diameter (mm), at the planned cut line

*CI* confidence interval, *PDAC* pancreatic ductal adenocarcinoma, *IPMN* intraductal papillary mucinous neoplasm, *BMI* body mass index, *MPD* main pancreatic duct, *PPPD* pylorus-preserving pancreaticoduodenectomy, *SSPPD* subtotal stomach-preserving pancreaticoduodenectomy, *POPF* postoperative pancreatic fistula, *BL* biochemical leak, Statistical analysis: Mann- Whitney U test for contentious variables. χ2 analysis for discrete variables

As shown in Table 3, the absence of combined PV resection (p = 0.002) and soft pancreas (*p* = 0.001) were selected as significant intraoperative risk factors for POPF according to univariate analysis, and only soft pancreas (*p* = 0.050) was calculated as an independent risk factor for POPF.

**Table 3:** Univariate and multivariate analysis for evaluating intraoperative risk factors associated with POPF

Variables	non-POPF, BL (*n* = 235)	POPF Grade BC (*n* = 27)	*P*-value (Univariate)	Odd’s ratio (95% CI)	*P*-value (Multivariate)
Procedure: PPPD/SSPPD/PD	6/203/26	0/25/2	0.577		
Laparoscopic surgery (yes/no)	9/226	3/24	0.087		
**Combined PV resection (yes/no)**	**107/128**	**4/23**	**0.002**	2.72 (0.80–9.31)	0.110
Combined artery resection (yes/no)	9/226	3/24	0.087		
Combined distal pancreatectomy (yes/no)	3/232	1/26	0.330		
**Pancreatic texture judged intraoperatively (soft/hard)**	**102/133**	**21/6**	**0.001**	**2.89 (1.00–8.35)**	**0.050**
Diameter of main pancreatic duct judged intraoperatively	4 (1–15)	3 (2–8)	0.054		
Pancreatic stent (yes/no)	123/112	17/10	0.654		
Operation time (min)	498.5 (286-1373^a^)	496.0 (333–670)	0.591		
Intraoperative blood loss (ml)	713.0 (20-20983^b^)	692.0 (210–5522)	0.234		

^a^This case underwent SSPPD, transverse colectomy and low anterior resection for triple cancer (duodenal papilla, transverse colon and rectum)

^b^This case developed intraoperative massive bleeding due to the presence of intraabdominal abscess and severe adhesion to adjacent organs and vessels, but finally recovered

*CI* confidence interval, *PPPD* pylorus-preserving pancreaticoduodenectomy, *SSPPD* subtotal stomach-preserving pancreaticoduodenectomy, *PD* pancreaticoduodenectomy, *PV* portal vein, *POPF* postoperative pancreatic fistula, *BL* biochemical leak, Statistical analysis: Mann- Whitney U test for contentious variables. χ2 analysis for discrete variables

### Risk categorization of POPF according to the pancreas-visceral fat CT value ratio and pancreatic outer contour

**Fig. 4:**
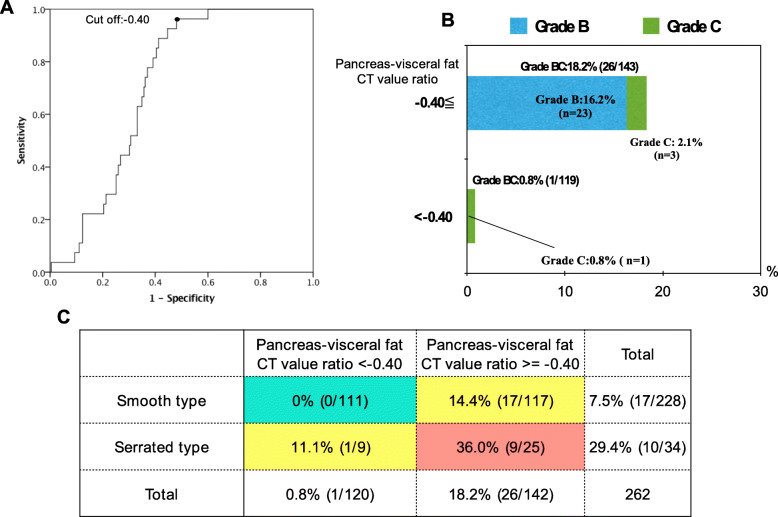
Prediction of POPF in total 262 patients. **a**. Receiver operating characteristic (ROC) curve. Cut-off point of pancreas-visceral fat CT value ratio is − 0.40 (AUC:0711). **b.** The incidence of POPF according to the pancreas-visceral fat CT value ratio. **c**. The 2 × 2 Contingency table analysis for the incidences of POPF according to pancreas-visceral fat CT value ratio and pancreatic outer contour POPF: postoperative pancreatic fistula

First, to clarify the clinical relevance of the pancreas-visceral fat CT value ratio for predicting POPF after PD, the optimal cut-off point was determined using a receiver operating characteristic curve (ROC). As shown in Fig. 4a, the cut-off point of the pancreas-visceral fat CT value ratio was − 0.40 (AUC: 0.711). Moreover, the incidence of POPF grade B/C was 18.2% (26/143) in patients with a value of − 0.40 or greater, which was significantly higher than the incidence of 0.8% (1/119) in patients with a pancreas-visceral fat CT value ratio of less than − 0.40 (*P* < 0.001), as shown in Fig. 4b. Figure 4c shows the 2 × 2 contingency table analysis for predicting POPF patients divided based on the pancreas-visceral fat CT value ratio and pancreatic outer contour. The analysis revealed that the incidence of POPF grade B/C was markedly high (36.0%, 9/25) in patients with a pancreas-visceral fat CT value ratio of − 0.4 or greater and serrated-type contour, whereas it was 0% (0/110) in patients with a pancreas-visceral fat CT value ratio of less than − 0.4 and smooth-type contour. Therefore, the patients in whom PD was proposed could be categorized into the POPF high-risk group (pancreas-visceral fat CT value ratio > = − 0.40 and serrated type) or low-risk group (pancreas-visceral fat CT value ratio < − 0.40 and smooth type) according to these factors regardless of intraoperative pancreatic consistency.

### Association between pancreatic configuration and pancreatic texture judged intraoperatively

The incidence of serrated-type contour was significantly higher in patients with soft pancreas than in patients with hard pancreas (22%, 27/123 vs. 5%, 7/139. *p* < 0.001). On the other hand, the value of the pancreas-visceral fat CT value ratio tended to be lower in patients with soft pancreas than in patients with hard pancreas (median: − 0.40 vs. -0.38, *p* = 0.066).

Next, we focused on the association between the incidence of POPF and our predictors in only soft pancreatic patients because the intraoperative judgement of soft pancreas was the only significant factor predicting POPF. In the same manner as Fig. 4c, the 2 × 2 contingency table analysis for predicting POPF patients divided based on the pancreas-visceral fat CT value ratio and pancreatic outer contour was conducted only for soft pancreatic texture. The analysis revealed that the incidence of POPF grade B/C was markedly high (45.0%, 9/20) in patients with a pancreas-visceral fat CT value ratio of − 0.4 or greater and serrated-type contour, whereas the incidence was 0% (0/57) in patients with a pancreas-visceral fat CT value ratio of less than − 0.4 and smooth-type contour (Table 4), proving that even patients with soft pancreas should be categorized into POPF high- (Table 4, red), intermediate- (Table 4, yellow) and low- (Table 4, green) risk groups according to the preoperative CT scans.

**Table 4:**
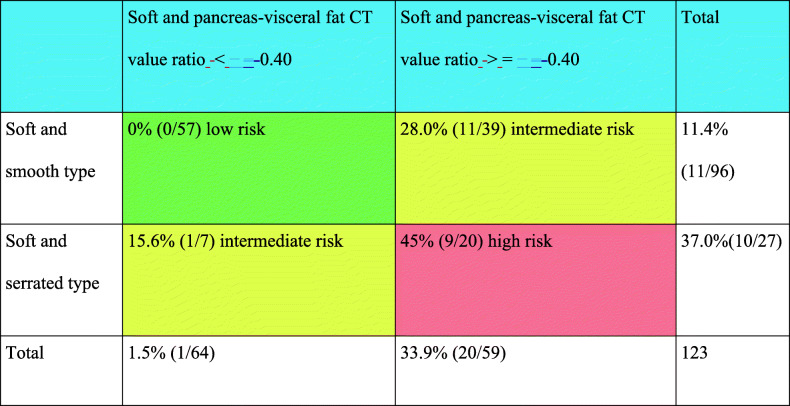
The 2 × 2 Contingency table analysis for the incidences of POPF according to pancreas-visceral fat CT value ratio and pancreatic outer contour in the 123 patients with soft pancreatic texture

### Histological evaluation of the pancreatic stump to estimate the percentage of parenchymal and interlobular (PI) area

**Fig. 5:**
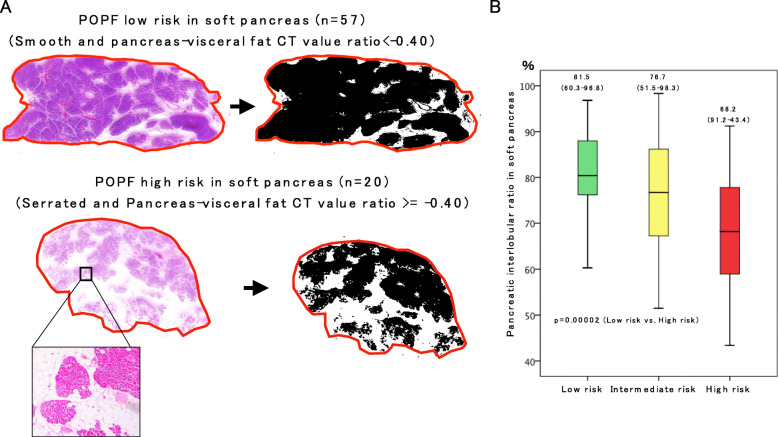
Histological evaluation of the pancreatic stump to estimate the percentage of a parenchymal and interlobular (PI) area using ImageJ software. **a.** Loupe images of the pancreatic stump with hematoxylin and eosin staining and their binary images by ImageJ software. After the outer circumference of the entire cut surface (red line) is manually outlined, the entire cut surface area is measured by using ImageJ software. The black area is regarded as the PI area. The white area is regarded as the area including fatty tissue. Magnified pictures showed representative images according to the POPF low or high-risk groups in the soft pancreas. In a typical case with POPF low risk (upper picture of A), the percentage of PI area/entire surface area was 80.0% (252.1 / 315.8 × 100). On the other hand, in a typical case with POPF high risk (lower picture of A), the percentage of PI area/entire surface area was 52.6% (132.6 / 252.1 × 100). **b.** Box plot graph for the comparison of the percentage of PI area. It is significantly higher than in the POPF low-risk group than that of the high-risk group (*p* = 0.00002)

To confirm whether our risk categorization based preoperative CT configurations reflects the quality of the pancreas, loupe images of the pancreatic stump with haematoxylin and eosin staining were analysed in soft pancreatic patients by using their binary images with ImageJ software [27] in an attempt to estimate the percentage of the PI area and the degree of fat infiltration. In soft pancreatic patients with a high risk of POPF (*n* = 20), the percentage of the PI area was significantly lower than that in patients with a low risk of POPF (*n* = 57) (68.2 vs 81.5%, *p* = 0.00002) **(**Fig. 5**)**.

## Discussion

In the present study, using a homogeneous cohort of patients who underwent pancreaticojejunostomy with PWST, we newly revealed that the pancreas-visceral fat CT value ratio, serrated-type pancreatic contour, and a history of chemoradiotherapy were strong preoperative predictors of POPF after PD. Furthermore, soft pancreatic texture was selected as the only intraoperative risk factor for POPF, and this result has been widely accepted among pancreatic surgeons until now. Among these risk factors, we considered that the pancreas-visceral fat CT value ratio and serrated-type pancreatic contour were closely associated with the degradation of pancreatic parenchymal quality characterized by parenchymal fat deposition, which was evidenced by histological evaluation of the parenchymal stump.

Since the effective management of POPF has proven to be a difficult challenge despite recent improvements in postoperative patient care, the early identification of POPF high-risk groups has made a paradigm shift among pancreatic surgeons from a reactive and passive approach that begins to treat POPF when it becomes apparent to a proactive approach that depends on early anticipation and timely prevention through prophylactic measures. However, this approach is predicated on the assumption that POPF high-risk groups can actually be predicted. To predict POPF more precisely, a clinical scoring system by Callery MP et al. [28] was considered to be very useful because the incidence of clinically relevant POPF reached more than 67% in patients with scores greater than seven, which consisted of gland texture, pathology (pancreatic adenocarcinoma or pancreatitis or others), pancreatic duct diameter and the amount of intraoperative blood loss. However, this score cannot be assessed preoperatively because the scoring systems include several intra- and postoperative variables, such as gland texture, pathological diagnosis and blood loss. In particular, as pancreatic gland texture is judged by the surgeon’s hand intraoperatively, this approach is a very subjective method; therefore, preoperative indicators for predicting POPF should be identified as an alternative to conducting the proactive approach. When we treat high-risk groups of patients, we should pay much more attention to the occurrence of POPF from the preoperative setting so that preoperative, careful informed consent can be provided to patients and their families. Postoperatively, we should carefully check the amylase levels of the drainage discharge and the appearance of fluid. If inflammatory reactions are unexpectedly escalated or continue in high-risk groups, an early follow-up CT scan should be conducted to detect the presence or absence of pancreatic fistula.

Among the various preoperative risk factors, the pancreas-visceral fat CT value ratio was selected as the most independent factor for predicting POPF rather than the CT value of the pancreatic parenchyma itself. We considered that the pancreas-visceral fat CT value ratio represents the quality of the pancreatic parenchyma. Kitajima Y et al. [29] measured intramuscular adipose tissue content (IMAC) using CT, and this measure has recently attracted much attention for evaluating the quality of skeletal muscle because several studies have revealed that increased IMAC is positively linked to worse survival after resection of PDAC [30] and to an increased complication rate after hepatectomy for hepatocellular carcinoma [31]. Nevertheless, there have been no reports regarding pancreatic parenchymal quality. In this study, we hypothesized that a lower quality of the pancreatic parenchyma might result in vulnerability of pancreaticojejunal anastomosis due to severe fat infiltration. Since IMAC is calculated by the ratio of the multifidus muscle to the subcutaneous fat CT attenuation value, we analysed whether the pancreas-visceral fat CT value ratio represented the quality of the pancreas by comparing the incidence of POPF, and this is the first report regarding the estimation of pancreatic parenchymal quality using plain CT images.

A history of chemoradiotherapy reduced the incidence of POPF in the present study. In general, preoperative chemoradiotherapy is considered to reduce POPF after pancreatectomy because radiation induces intralobular fibrosis and exacerbates its exocrine function [32, 33]. Moreover, in our institution, most candidates who undergo preoperative chemoradiotherapy are patients with advanced PDAC, in whom exocrine function is generally ruined due to MPD obstruction. As a result, preoperative chemotherapy significantly reduced POPF after PD in our cohort.

Serrated-type pancreatic contour determined by preoperative plain CT was also selected as another independent risk factor for POPF. In fact, the incidence of POPF was significantly higher in patients with a serrated-type pancreas (29.4%, 10/34) than in patients with a smooth pancreas (7.4%, 17/228). Indeed, serrated-type contour was mostly seen in patients with soft pancreas, whereas this type of contour was barely found in patients with a hard pancreatic texture. The precise aetiology of significant changes in the irregularity of the borders of the pancreas is unknown, but serrated-type pancreatic contour has been reported to be the result of ageing or acute weight loss after the reversal of type 2 DM treated by a low-calorie diet [25]. When we focused on basic research regarding pancreatic exocrine architectures, ghrelin, a hunger-stimulating hormone produced by the fundus of the stomach, increased exocrine pancreatic fractal dimensions and textural entropy and decreased the lacunarity of the acinar cell architecture in rats, regardless of age [34]. In the clinical setting, Sasaki K et al. [35] reported that the individual ghrelin ratio (POD1/prior to operation) was significantly lower in patients who developed complications, especially POPF and intraabdominal abscess, than in those who did not. The lack of ghrelin exertion and weight loss in patients with type 2 DM might be related to the formation of a serrated-type pancreas and its parenchymal frailty, which significantly accelerate the incidence of POPF. In our present study, however, the incidence of a serrated-type pancreas was comparable between diabetic and non-diabetic patients, and the level of ghrelin in the blood was not measured. Therefore, further research is needed to elucidate the cause of serrated-type pancreatic contour.

Soft pancreas is generally characterized by a narrow pancreatic duct and vulnerable parenchymal texture, resulting in a high risk of POPF after PD. However, as shown in our 2 × 2 contingency table analysis (Table 4), the incidence of POPF grade B/C was 0% (0/57) in patients with a pancreas-visceral fat CT value ratio of less than − 0.4 and smooth-type contour, even though these patients were categorized as patients with soft pancreas intraoperatively. The high-risk group showed an obviously high incidence of POPF (9/20). Moreover, these pancreases were characterized by obvious fat infiltration pathologically, as shown in Fig. 5. Indeed, fatty pancreas is considered to be a high-risk factor for POPF, and Mathur A et al. [36] demonstrated that patients with increased fat and decreased fibrosis had a higher risk of POPF after PD. Gaujoux S et al. [37] also showed that an increased body mass index, fatty pancreas, and the absence of fibrosis were associated with a risk of POPF after PD. In these reports, the amount of fat deposition and degree of fibrosis were examined by histological findings, and therefore, these factors could not be assessed preoperatively. To assess the amount of pancreatic fat and its influence on POPF preoperatively, Lee SE et al. [38] suggested the usefulness of relative signal intensity decreases in preoperative dual-gradient-echo magnetic resonance imaging (MRI) since preoperative measurements of pancreatic fat by MRI offer noninvasive prediction of the occurrence of POPF. In an attempt to predict pancreatic texture preoperatively, Kuwahara T et al. [24] analysed the usefulness of EUS-EG in 59 patients, 26 with soft pancreas and 33 with hard pancreas, and revealed that the mean elasticity of the pancreas measured by EUS-EG (> 70) was an independent predictor of POPF. Although MRI and EUS-EG are considered useful tools, the usage of these modalities is still limited for the preoperative assessment of PD; therefore, our predictors measurable by plain CT are considered feasible and reasonable for predicting POPF.

**Fig. 6:**
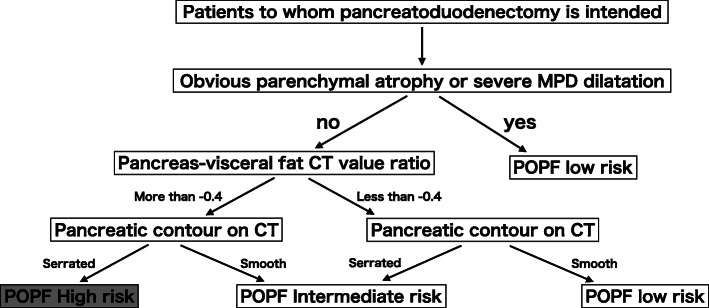
Flow chart for determining the POPF risk category. POPF: postoperative pancreatic fistula MPD: main pancreatic duct

Taken together, the high pancreas-visceral fat CT value ratio, serrated-type pancreas and these combinations could be associated with the infiltration of adipose tissue into the pancreas, resulting in parenchymal frailty. The frailty of the parenchyma makes it difficult to perform pancreaticojejunostomy and induces the vulnerability of anastomosis regardless of the surgeon’s anastomosis technique. Therefore, we established a new strategic schema (Fig. 6) for the precise prediction of POPF preoperatively, which might contribute to the proactive approach for POPF.

## Conclusions

The pancreas-visceral fat CT value ratio > = − 0.40 and serrated-type pancreas allowed us to preoperatively identify a true POPF high-risk group regardless of pancreatic texture. Preoperative identification of a POPF high-risk group enabled us to develop a proactive strategy, such as the administration of somatostatin analogues and early follow-up CT scans for preventing POPF or its aggravation and for early detection. To establish the new strategy for preventing POPF for high-risk patients is imperative to improve a surgical outcome of PD.

## Data Availability

The datasets generated and/or analyzed during the current study are not publicly available due to the data is confidential patient data but are available from the corresponding author on reasonable request.

## Abbreviations

POPF: Postoperative pancreatic fistula
PD: Pancreatoduodenectomy
PWST: Pair-watch suturing technique
MPD): Main pancreatic duct
CT: Computed tomography
EUS-EG: Endoscopic ultrasonography-elastography
DM: Diabetes mellitus
PPPD: Pylorus-preserving PD
POD: Postoperative day
BL: Biochemical leak
ROIs: Regions of interest
BMI: Body mass index
PV: Portal vain
ROC: Receiver-operating characteristic
IPMN: Intraductal papillary mucinous neoplasm
HU: Hounsfield Unit
PDAC: Pancreatic ductal adenocarcinoma
PI area: Parenchymal and interlobular
IMAC: Intramuscular adipose tissue content
MRI: Magnetic resonance imaging
